# Supplementary figures and images for: The impact of atlas-based MR attenuation correction on the diagnosis of FDG-PET/MR for Alzheimer’s diseases— A simulation study combining multi-center data and ADNI-data
Source: PLoS One. 2020 Jun 3;15(6):e0233886. doi: 10.1371/journal.pone.0233886 (PMC7269241; doi:10.1371/journal.pone.0233886)

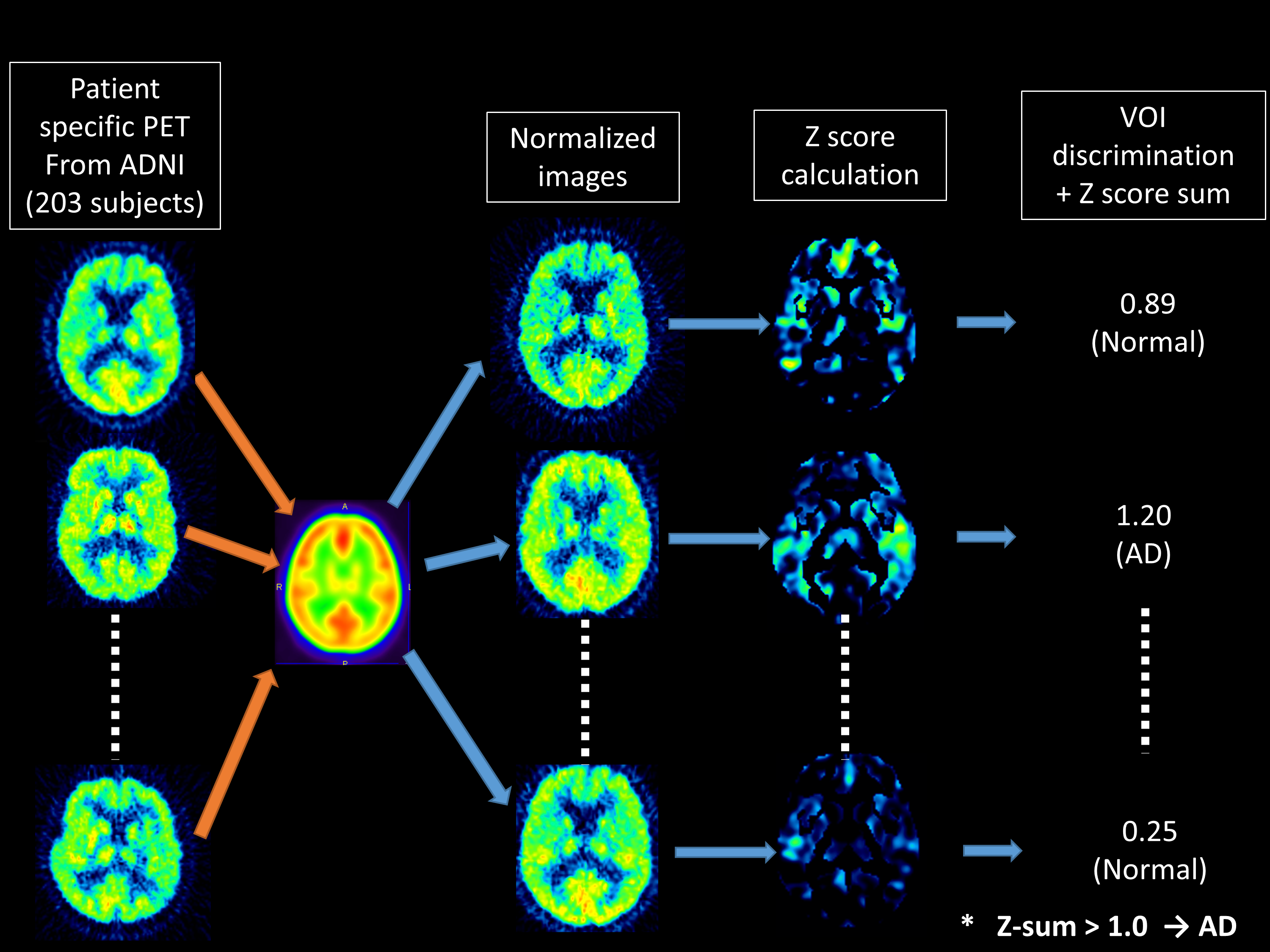

Supplement: S1 Fig — A detailed description is provided in the materials and methods section. (TIF) [file pone.0233886.s001.tif]

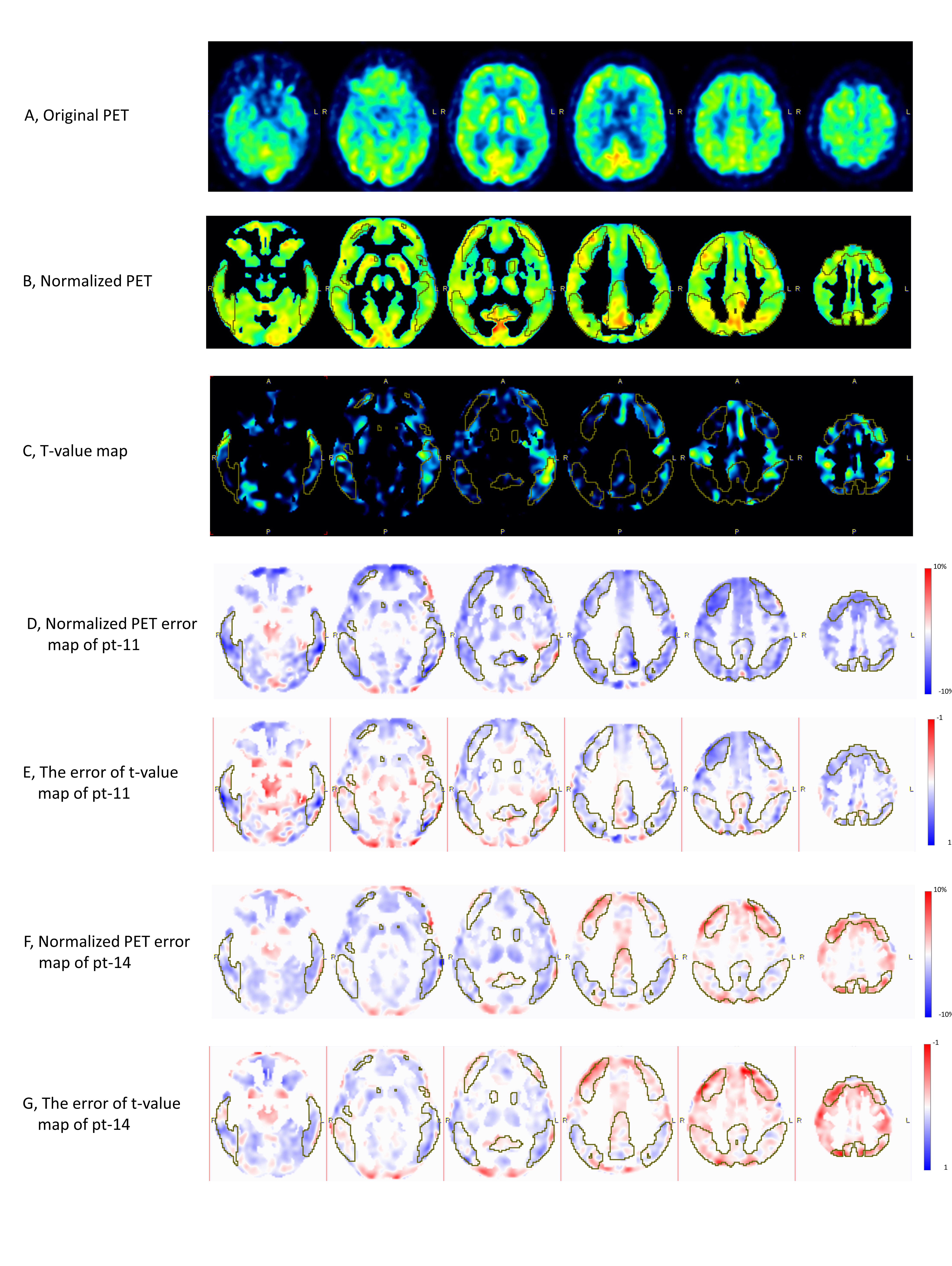

Supplement: S2 Fig — The original PET images extracted from ADNI-cohort (A) were spatially normalized to the SPM99 PET template, followed by smoothing with a Gaussian filter of 12 x 12 x 12 mm. Secondly, voxel values were normalized by dividing each image voxel value by the mean voxel value, averaged within a mask representing voxels with AD-preserved activity (B). The voxel-wise differences between the expected and patient-specific images are used to calculate t-values (C). The error map was obtained by dividing PET based on Atlas-AC by that on CT-AC, followed by the normalization to the identical space as spatially normalized ADNI-PET data. The original error map is shown in figures (D) and (F). We multiplied the normalized PET (B) with each normalized error map (D and F), followed by calculation of the t-value map of the simulated PET (not shown in this figure). The error of the t-value maps was obtained by subtracting the original t-value map from the simulated t-value map (E and G). The yellow VOI corresponds to AD-related voxels. The original PET-score of ADNI-data was 0.9385. The PET-score of simulated data was 1.0478 for pt-11 and 0.9497 for pt-14. More detailed figures are shown in S2 Fig. (JPG) [file pone.0233886.s002.jpg]
